# Selective binding of facial features reveals dynamic expression fragments

**DOI:** 10.1038/s41598-018-27242-2

**Published:** 2018-06-13

**Authors:** Charlotte Harrison, Nicola Binetti, Isabelle Mareschal, Alan Johnston

**Affiliations:** 1 Department of Experimental Psychology, University College London, London, UK; 2 Experimental Psychology, School of Biological and Chemical Sciences, Queen Mary University of London, London, UK; 3 School of Psychology, University of Nottingham, Nottingham, UK

## Abstract

The temporal correspondence between two arbitrarily chosen pairs of alternating features can generally be reported for rates up to 3–4 Hz. This limit is however surpassed for specialised visual mechanisms that encode conjunctions of features. Here we show that this 3–4 Hz limit is exceeded for eye gaze and eyebrow pairing, but not for eye gaze and mouth pairing, suggesting combined eye and eyebrow motion constitutes a dynamic expression fragment; a building block of superordinate facial actions.

## Introduction

Theories about the perceptual primitives that underlie the internal representation of dynamic facial expressions have typically been derived from observations of the coordinated actions of the musculature and jaw^1^ or the statistics of changes in appearance^2^. More recently, neurophysiological evidence has accrued that points to a hierarchy of face processing areas in the monkey and human temporal lobe^3^. The middle face patch has cells which are responsive to various multiple facial features and their interrelationships^4^ and the middle dorsal (MD) and anterior fundus (AF) face patches in the superior temporal sulcus have been identified as sensitive to facial motion^5^. This suggests the temporal lobe contains the machinery by which elementary facial features that underlie expressions are brought together. However, we currently lack an understanding of whether the coordinated actions that generate a particular expression are perceived as a set of independent dynamic features or whether they are bound holistically into a perceptual group.

The temporal ceiling on our ability to judge feature correspondence across two synchronously alternating feature sets is remarkably consistent, around 3 Hz, regardless of whether the features come from the same or different categories or modalities^6^. It is suggested that this limit results from temporal constraints on a generic perceptual routine or control process that has to identify and store some feature and then detect the corresponding contiguous feature in the companion sequence^7^. If the alternations occur too quickly the temporal relationship becomes unclear and mismatches arise^8,9^. If the correct feature correspondence can be reported at higher alternation rates, it indicates the features are jointly available in a specialised system and they alternate as a coupled unit^6,10^. We can use this phenomenon to study dynamic feature grouping in face perception.

Note first that the relationship between the direction of motion of two stimuli moving along the same motion axis can be correctly determined at a high alternation rate (10–20 Hz) even for large retinal separation, however the temporal limit drops to around ~3–4 Hz when the motion signals are orthogonal to one another^11^ in a T configuration, suggesting orthogonal motion signals are processed by separate mechanisms at the global motion level. In the current study, we used orthogonal avatar facial actions to avoid low-level motion grouping.

## Results

**Figure 1:**
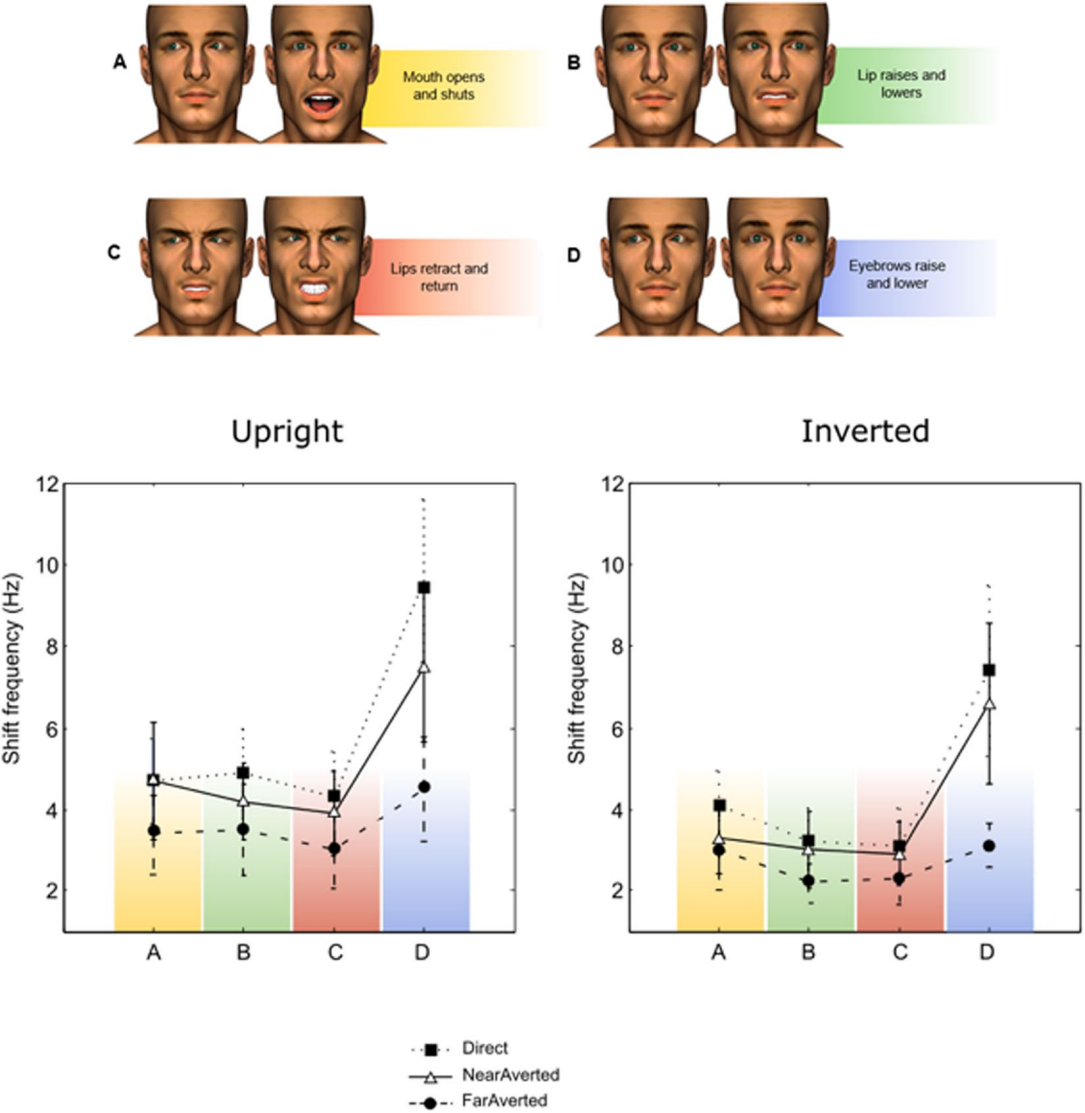
Illustrates the type of facial movement for each condition (**A**–**D**); the eye movements in each case could be between left averted and right averted (near or far) or averted and direct (direct). The graphs show the mean limiting frequency at which participants still performed above chance in each condition of the experiment, for upright and inverted faces (error bars: ± 1*SE*).

We asked whether moving facial features are processed separately or as dynamic feature conjunctions. We chose four facial actions to pair with left and right eye movement in four separate experiments (Fig. 1, top). The avatar’s eyes moved in one of three ways to assess the influence of direct gaze as compared to averted gaze; (1) averted to direct (direct), (2) averted to averted (far) and (3) averted to averted (near). Participants viewed two static images that alternated at varying rates (1, 2, 3, 4, 6, 10, 14 Hz). They were asked to report the direction of the eyes when the mouth was open or eyebrows were raised (a binding task). Typically, participants could perform the task at slow alternations but were at chance for 14 Hz alternations. The data were fitted with a Weibull function, and the rate at which participants were performing the task significantly above chance (62.5%; 40 trials/point) was extracted for analysis. The first experiment (A) paired eye gaze with mouth opening. In the three additional experiments, we paired eye gaze with (B) upper lip motion and (C) upper and lower lip retraction with furrowed eyebrows to assess the effect of emotional valence (disgust and anger respectively) and with (D) eyebrow motion as an alternative vertical feature motion.

The data are shown in Fig. 1. The temporal rate limit was significantly faster for upright compared to inverted stimuli (*F*(1,35) = 50.61, *p* < 0.001, *η*_*p*_^*2*^ = 0.59) which indicates that the task engages face specific processing rather than just low-level motion mechanisms. There was a significant main effect of experiment (*F*(3,35) = 13.7, *p* < 0.001, *η*_*p*_^*2*^ = 0.54). Pairwise comparisons of the overall means showed significant differences in the binding limit between the Eyebrow Raised experiment (D: *M* = 6.51, *SE* = 0.39) and the Open Mouth (A: *M* = 3.9, *SE* = 0.39; *p* < 0.001), Raised Lip (B: *M* = 3.56, *SE* = 0.4; *p* < 0.001) and Retracted Lip (C: *M* = 3.31, *SE* = 0.42; *p* < 0.001) experiments. No other significant differences between the experiments were found. The results indicate that participants could perform at above chance levels at significantly faster speeds in the Eyebrow Raised experiment than the others. There was a significant main effect of gaze type (*F*(2,70) = 21.22, *p* < 0.001, *η*_*p*_^*2*^ = 0.38). Pairwise comparisons of the overall means showed there was a significant poorer performance in the Far Averted (*M* = 3.1, *SE* = 0.13) than in the Direct (*M* = 5.22, *SE* = 0.33; *p* < 0.001) and Near Averted (*M* = 4.56, *SE* = 0.32; *p* = 0.002) gaze conditions, which did not differ (*p* = 0.053). There was also a significant interaction between experiment and gaze type (*F*(6,70) = 4.13, *p* = 0.001, *η*_*p*_^*2*^ = 0.26), indicating this difference was larger in the Eyebrow Raised experiment. One explanation for the poor performance in the Far Averted condition is a break down in the ability to perceive the shift as apparent motion. The ceiling for seeing apparent motion at high alternation rates is lower for larger displacements^12^.

## Discussion

Direct gaze did not confer any advantage over near averted gaze, indicating direct gaze did not pair preferentially with any other feature configuration. The presence of emotional expressions such as anger (C, exposed teeth) or disgust (B, raised top lip) did not confer any advantage in judging temporal contiguity and consequently this implies the judgement of their temporal relationship with eye gaze falls on generic perceptual routines. Eye gaze direction and emotional expression have been shown to interact in other paradigms. For example, direct gaze paired with a moderately angry face and averted gaze paired with a moderately fearful facial expression have been shown to increase activation in the amygdala^13,14^ and lead to quicker emotional valence classification^15^. This interaction has been explained though the concept of shared signals in that both direct gaze and anger and averted gaze with fear signify threat^16^. However, an appreciation of threat requires a cognitive appraisal that is unlikely to modulate at high temporal frequencies.

We found that temporal correspondence could be determined at significantly faster rates for eye gaze paired with raised eyebrows than when the eye gaze was paired with mouth movements, which was limited to the typical rate of around 3–4 Hz, characteristic of the general perceptual routine for comparing the timing of feature alternations. The task is not simply limited by low-level motion cues since we observed a lower binding limit for upright relative to inverted faces overall and for experiment D considered separately (*F*(1,9) = 7.40, *p* = 0.024). The eyebrows are closer to the eyes than the mouth, however we can discount the idea that the spatial proximity of the eye and eyebrow facilitate temporal binding. There is no evidence that spatial proximity of itself can enhance feature binding. Maruya *et al*.^11^ showed that separating moving features to be compared in the T configuration between 10 and 40 degrees of visual angle had little effect on the critical frequency. At smaller separations (2–12 degrees) Rainville and Makous^17^ report little effect of separation on synchronous vs asynchronous motion discrimination. Significantly, Holcombe and Cavanagh^10^ found participants could correctly report the configuration of overlapping red leftward slanted and green rightward slanted gratings that alternated at high rates (18 Hz) but they could not say whether a leftward slanted grating was contiguous with a spatially abutting red patch in a similarly alternating sequence, unless the alternation rate was below 3–4 Hz. The key difference here is not the spatial proximity of the features, but that a coloured oriented grating is processed as a perceptual unit.

The enhanced correspondence rate for eye gaze and eyebrow position of around 8 Hz for upright faces, well about the generic 3–4 Hz limit, implicates conjunction coding for dynamic variation in the region of the eye. Eye gaze direction and eyebrow position are coded as a perceptual unit. The more general implication of this result is that we have special perceptual mechanisms for the encoding of expression fragments that form the building blocks of superordinate facial actions. This paradigm promises the discovery of further perceptually grounded facial action units complementing schemes based on coordinated groups of muscle actions^1^.

## Methods

### Participants

Each experiment used separate groups of participants. In Experiment A, ten participants, nine of which were female, participated (*M* = 21.3, *SD* = 3.55, range = 18–26 years). In Experiment B there were ten participants, eight of which were female (*M* = 19.9, *SD* = 2.21, range = 18–20 years). In Experiment C, there were nine participants, seven of which were female (*M* = 24.8, *SD* = 5.08, range = 18–35 years). One participant was rejected as the Weibull function provided a poor fit to the data. Finally, in Experiment D, there were ten participants, nine of which were female (*M* = 21.1, *SD* = 1.47, range = 18–26 years). Written informed consent was obtained from participants prior to the experiment. Ethical approval for the study was obtained from the UCL Experimental Psychology Departmental Ethics Committee CPB/2010/003. All procedures adhered to the guidelines of the Declaration of Helsinki.

### Stimuli and Display

The experimental programs were written in Matlab (Version 2013b, Mathworks; http://www.mathworks.co.uk/) using the PsychToolbox extension^18^ and stimuli were presented on a Mitsubishi Diamond Plus 230 SB CRT monitor. The screen resolution was 1280 × 1024 and the refresh rate was 60 Hz. Participants gave responses using a standard computer keyboard. Avatars were rendered using Poser Pro 9 software (SmithMicro; http://poser.smithmicro.com) and adjusted in Photoshop CS6 Extended (Adobe; http://www.adobe.com). The screen was viewed from 57 cm and the head subtended 10.7 by 8.1 degrees of visual angle (dva).

Horizontal movement was created by a change in gaze direction; these changes in gaze were consistent across all four experiments. Direct gaze shifts moved from leftwards at 70° rotation to straight ahead, near averted gaze shifts were from 35° of rotation left to 35° right, and far averted gaze shifts were from 70° of rotation left to 70° right. The extent of movement in the image corresponded to either 0.2 dva or 0.4 dva in the case of far averted to far averted. In all four experiments both upright and inverted faces were presented to test for the presence of specialised facial processing.

#### Experiment A

**Figure 2:**
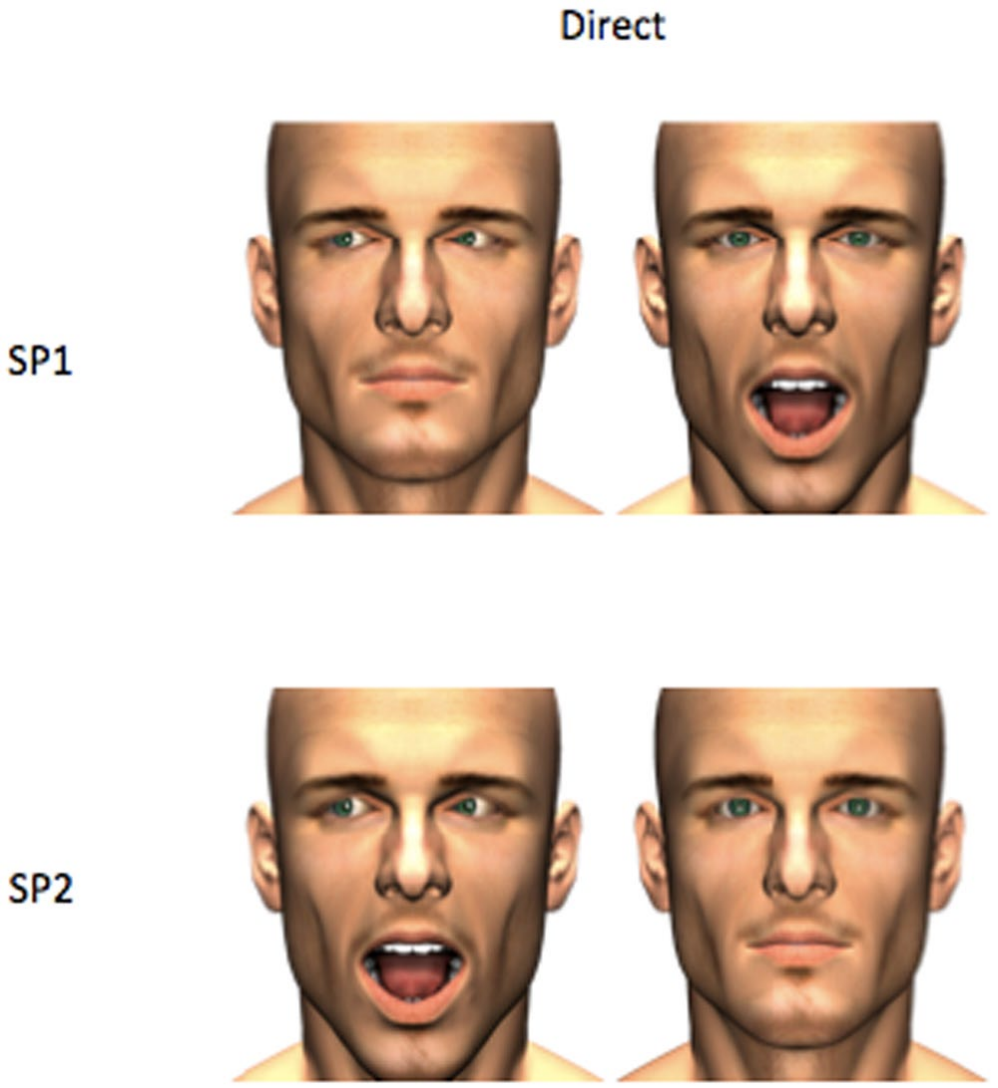
The two potential starting points (SP) on direct gaze trials, and the image with which it alternated.

The vertical movement resulted from the mouth opening and closing. There were two potential starting configurations (Fig. 2). The starting frame could either have eyes rotated 70° left and mouth shut (SP1) or eyes rotated 70° left and mouth open (SP2). In this experiment participants were asked, in which direction were the eyes facing when the mouth was open.

#### Experiment B

The vertical movement arose from the rise and fall of the upper lip. This time, participants were asked, in which direction were the eyes facing when the lip was raised.

#### Experiment C

The vertical movement was created by both the upper and lower lips retracting and returning. In addition the eyebrows were lowered to help create an angry expression. The eyebrows were fixed throughout. In this experiment, participants were asked, in which direction were the eyes facing when the mouth was open.

#### Experiment D

In the final study, the vertical movement was created by the rise and fall of the eyebrows. The ‘lower’ point was a neutral expression. In this experiment, participants were asked, in which direction were the eyes facing when the eyebrows were raised.

### Experimental Design

In all experiments, gaze type (direct vs. close averted vs. far averted) and orientation (upright vs. inverted) were blocked separately, giving six different block types. Stimuli were presented at one of seven different alternation rates (1, 2, 3, 4, 6, 10, 14 Hz) on each trial, for a total duration of three seconds. There were two potential starting positions for the stimuli. Participants had to identify which of the two alternative sequences had been presented. Correct answers for each starting point were combined for analysis. Participants completed each block type twice, meaning they saw each trial type 40 times. The proportion of correct trials were plotted as a function of alternation rate for each subject and the Weibull function was fit to the data. We recovered the point on the fitted function corresponding to the frequency at which participants responded better than chance at the 0.05 level as calculated from the binomial distribution which was 62.5%. This data was then subject to a 3-way mixed effects design ANOVA. Comparisons of the reported simple main effects were Bonferroni corrected.

The datasets generated during and/or analysed during the current study are available from the corresponding author on reasonable request.
